# Blood, Toil, and Taxoteres: Biological Determinants of Treatment-Induced ctDNA Dynamics for Interpreting Tumor Response

**DOI:** 10.3389/pore.2022.1610103

**Published:** 2022-05-19

**Authors:** Christopher T. Boniface, Paul T. Spellman

**Affiliations:** ^1^ Knight Cancer Institute, Oregon Health & Science University, Portland, OR, United States; ^2^ Cancer Early Detection Advanced Research Center, Knight Cancer Institute, Oregon Health & Science University, Portland, OR, United States

**Keywords:** biomarkers, liquid biopsy, circulating tumor DNA, ctDNA, tumor growth, treatment response

## Abstract

Collection and analysis of circulating tumor DNA (ctDNA) is one of the few methods of liquid biopsy that measures generalizable and tumor specific molecules, and is one of the most promising approaches in assessing the effectiveness of cancer care. Clinical assays that utilize ctDNA are commercially available for the identification of actionable mutations prior to treatment and to assess minimal residual disease after treatment. There is currently no clinical ctDNA assay specifically intended to monitor disease response during treatment, partially due to the complex challenge of understanding the biological sources of ctDNA and the underlying principles that govern its release. Although studies have shown pre- and post-treatment ctDNA levels can be prognostic, there is evidence that early, on-treatment changes in ctDNA levels are more accurate in predicting response. Yet, these results also vary widely among cohorts, cancer type, and treatment, likely due to the driving biology of tumor cell proliferation, cell death, and ctDNA clearance kinetics. To realize the full potential of ctDNA monitoring in cancer care, we may need to reorient our thinking toward the fundamental biological underpinnings of ctDNA release and dissemination from merely seeking convenient clinical correlates.

## Background

Circulating tumor DNA (ctDNA) is extracellular DNA in plasma that originates from tumor cells and has emerged as a useful biomarker in non-invasive liquid biopsy (1-3). ctDNA abundance shows broad correlation with tumor burden and generally reflects the tumor DNA content such that clinical assays are commercially available for detection of molecular/minimal residual disease (MRD) and tumor mutational profiling (4–6). However, there are currently no ctDNA-based assays approved for serial monitoring during treatment to assess immediate tumor response and treatment efficacy.

Serial ctDNA monitoring during treatment can provide insight into underlying biological factors that can potentially be used to predict response, treatment efficacy, and long-term outcomes (7–11). In practice however, ctDNA levels can appear erratic across time points and are often inconsistent between patients with similar disease and treatment. This variability may be partially the result of disparate sampling frequency (often within the same study), extraction methods, and analytical approaches between studies. More likely, this variation is driven by factors that have yet to be elucidated and may vary between patients, such as individual host physiology, tumor location, tumor biology, and treatment modality.

Evidence that ctDNA concentration is more dependent on tumor cell replication rates than simply on tumor volume also suggests that understanding tumor biology and patient physiology are necessary to guide proper interpretation of ctDNA dynamics (12, 13). In some studies, early spikes in ctDNA shortly after treatment may predict a favorable clinical response, in keeping with the hypothesis that shedding is directly associated with treatment-induced tumor cell death (14–16). It is unclear however, how soon after treatment initiation this spike must occur in such cases, emphasizing the importance of collection timing. Nevertheless, early and rapid ctDNA clearance during treatment has consistently been shown to correlate with objective response and outcome (17–20). Evidence supports the idea that ctDNA release is clearly a byproduct of tumor cell proliferation, though whether this is through increased cell turnover and higher death rates or active release during cellular expansion is still an open question.

This review seeks to discuss the biological sources of variability in ctDNA abundance, with the hope that thoughtful analysis and a mechanistic understanding of ctDNA release will allow improved approaches to ctDNA interpretation in clinical response and progression.

### A Note on Circulating Tumor DNA Detection and Its Implications for This Review

Typically, ctDNA is detected and characterized using methods such as droplet digital polymerase chain reaction (ddPCR) or next-generate sequencing (NGS) (reviewed by Heitzer et al. (2)) either targeting genomic positions based on *a priori* knowledge of tumor mutations or by calling mutations *de novo* at novel sites and recurrent hotspots. Although both *a priori* and *de novo* approaches to ctDNA detection assume that molecules harboring alternate alleles are tumor-derived, the later approach allows for ctDNA detection without *a priori* knowledge of tumor-specific mutations but is subject to much more uncertainty. The detection of tumor-derived copy-number aberrations in cell-free DNA is also possible and is dependent solely on read counts to detect gains or losses found in tumor cells (21). *De novo* mutations can be called from whole-genome and whole-exome sequencing, or smaller panels targeting just a few sites or genes known to harbor recurrent mutations. Panels intended to detect mutations at these canonical sites are often less informative about passenger mutations, secondary drivers, and subclonal populations. The same may be true for tumor-informed and patient-specific panels depending on the breadth of the panel used and the sampling bias of the original tumor tissue used to design the panel, particularly in the case of high intratumoral heterogeneity. These various approaches further confound our ability to compare results across studies, patient populations, and cancers. This problem is particularly true for studies where ctDNA was characterized by the prevalence of a single mutation in a single gene, where subclonal populations driven by other genetic aberrations may be under selective pressure during treatment. Of course, with a broader analytical space comes greater cost and complexity and additional challenges for implementation of accurate ctDNA assays in a clinical setting. For example, a simple ddPCR or amplicon-sequencing test to detect the presence of low-abundance EGFR mutations in the cell-free DNA of lung cancer patients is much cheaper and simpler to validate and execute in a diagnostic laboratory than a whole-exome or a multi-gene sequencing panel with similar accuracy and sensitivity. However, such an assay may not be representative of the entire tumor cell population, particularly if there are treatment-resistant subclones that harbor distinct genotypes. Consequently, careful evaluation of single-target vs. multi-target approaches is necessary.

Although the data available to assess ctDNA abundance as it relates to clinical observations, treatment response, and outcome consist primarily of mutant-allele detection and prevalence estimates, evidence suggests approaches like methylation profiling by whole-genome or targeted bisulfite sequencing may be more sensitive and are not dependent on the presence of genetic aberrations (5). A serial comparison of single-nucleotide variants (SNVs) and methylation profiles in EGFR T790M-positive advanced cancer patients found that methylation levels closely followed SNV mutant allele frequency and both were predictive of long-term treatment response (22). Silva et al. (23) reported changes in cell-free DNA methylation over time that were associated with therapy response and progression in prostate cancer patients. Few studies, however, have assessed methylation dynamics in cell-free DNA with high-frequency sample collection during early phases of treatment, and therefore the data presented here are biased toward somatic mutations as a means of ctDNA detection and characterization. It remains to be seen how broad the search space needs to be in order to effectively monitor tumor cell populations by ctDNA, and which reporter (i.e., mutations, methylation, etc.) will be the most informative, but may vary by patient, tumor, and treatment.

## Biological Factors That Most Affect Circulating Tumor DNA Abundance

**FIGURE 1:**
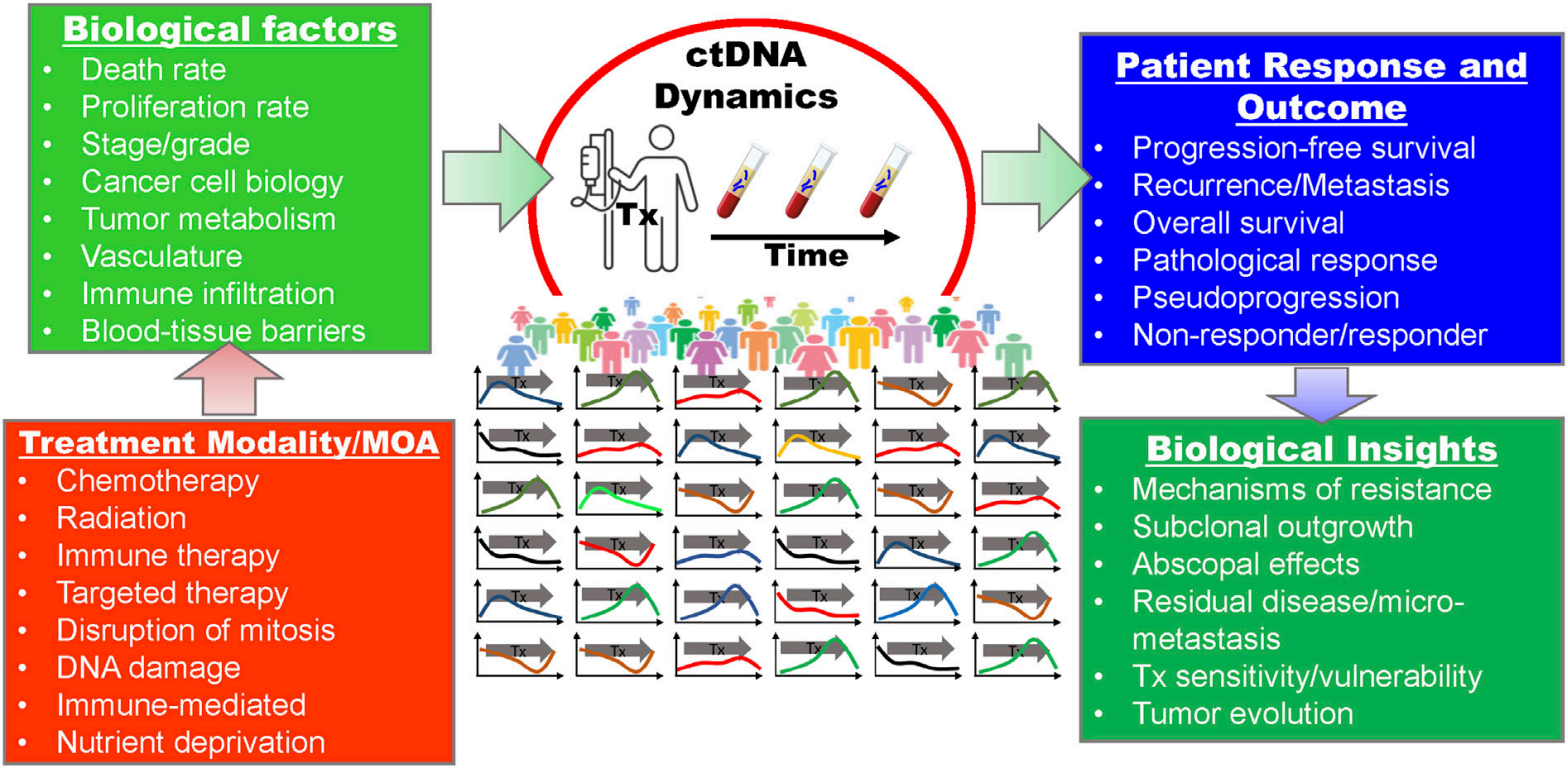
The relationships between treatment, biological factors, clinical indicators and outcomes, and potential insights with regard to serial ctDNA monitoring during treatment. MOA, mechanism of action; Tx, treatment.

In order to utilize ctDNA monitoring during treatment we must understand the various factors that impact ctDNA concentration over time. Multiple sources of ctDNA have been suggested including apoptosis, necrosis, and so called “active/passive release” (reviewed by Aucamp et al. (24)). Apoptosis, necrosis, and other forms of tumor cell death result in ctDNA release into interstitial space where it moves to the lymphatic system and blood circulation (8, 25). It is also hypothesized that extracellular DNA can be released from living cells in various contexts that are both energy-dependent and independent, and range in mechanism from shedding of mis-segregated DNA to intercellular signaling (26). Once ctDNA enters circulation, it is subject to further degradation by DNases in the blood and is putatively removed by the liver, spleen, and/or kidneys within 30–120 min (27). Changes in the balance of these processes due to treatment are assumed to be reflected in ctDNA dynamics, which, if correctly interpreted, may inform us about a patient’s disease state and response to therapy (Figure 1). Furthermore, disruption of biological homeostasis resulting from disease and treatment can increase overall levels of cell-free DNA, decreasing the relative abundance of ctDNA and thus impacting assay sensitivity. This can also make interpreting data from studies that simply report mutant allele frequency challenging without accounting for such changes in total cell-free DNA. The following sections address how the unique biology of a cancer and host physiology influences ctDNA abundance in the blood stream.

### Cancer Type and Biology

Even before our ability to distinguish ctDNA molecules amongst cell-free DNA, there appeared to be a clear relationship between cell-free DNA abundance and disease state. The more severe the patient’s cancer, the more cell-free DNA present in their blood (reviewed by de Miranda et al. (28), Grabuschnig et al. (26), Aucamp et al. (24)), suggesting that disease burden impacts cell-free DNA homeostasis. Later work has shown that ctDNA levels, independent of non-tumor, cell-free DNA, vary dramatically across disease type, tumor location, and stage (29–33). *In vitro* assays have helped isolate the mechanistic variables involved in cell-free DNA release, particularly with regard to known apoptotic and necrotic processes. These experiments found that cell-free DNA release can vary significantly between cell-lines with different phenotypes and histologies (34–39).

Observations in human cohorts also correlate ctDNA with tumor histology, grade, and stage. Various studies in neoadjuvantly-treated breast cancer patients found that ctDNA levels and mutations were significantly different between breast cancer subtypes (9, 11, 40, 41). Expression levels of the proliferation-associated nuclear protein, Ki-67, have also been directly associated with ctDNA characteristics in breast and lung cancers (42–44). Studies assessing ctDNA in non-small cell lung cancer (NSCLC) patients found that ctDNA concentration was correlated with tumor stage, histology, and degree of cytological atypia (45–47).

### Tumor Volume, Growth Rate, and Metabolism

Cell death has historically been considered the largest contributor to ctDNA and tumor volume the most reliable predictor of ctDNA abundance. More recently, various mechanisms and conditions have been proposed in which living tumor cells, particularly during mitosis, could shed DNA in both an energy-dependent and independent manner (26, 34). Therefore, it is likely the complex interplay between tumor cell proliferation and death that determines ctDNA abundance. This relationship has significant implications for how we should think about ctDNA measurements in the context of tumor volume, growth rate, and metabolism. For example, one can easily imagine a scenario where proliferation and death rates both increase but are in balance resulting in increased ctDNA shedding but no net change in tumor volume (48). Furthermore, although ctDNA may be hypothetically representative of the entire cancer cell population, it is likely subject to significant composition bias from differential cell turnover rates across subclones (3). Examining these variables *in vitro* and *in vivo* can shed light on which processes contribute more to ctDNA abundance.

Tumor growth rate and metabolism are often inferred by measuring tumor glucose uptake. Studies in metastatic melanoma patients found a strong correlation between the tumor PET avidity (a measure of cellular glucose uptake) and ctDNA abundance, independent of tumor volume (49). These results are supported by studies in resected NSCLC, where a correlation was found between increased mitotic rates and higher ctDNA levels measured 24 h prior to surgery, as well as increased levels of the proliferation marker Ki-67 (42–44, 47). Indeed, *in vitro* studies have consistently found that large amounts of cell-free DNA can accumulate in the media of actively proliferating cell populations independent of apoptosis or necrosis (34, 50–53). DNA fragments resulting from mis-segregation events during mitosis were found to be released by actively proliferating cancer cells via the creation of micronuclei (54, 55), but their relative contribution to overall ctDNA abundance *in vivo* remains unclear. Similar to the DNA products of necrosis, it has been assumed that these fragments would appear distinct from apoptotic ctDNA given their larger size and that their contribution would therefore be obvious. However, evidence is emerging that cleavage of larger DNA fragments by extracellular DNases may also occur (51). These DNA fragments might have an apoptotic fragmentation pattern, yet be generated by non-apoptotic mechanisms, such as release during proliferative states.

The relationship between tumor cell proliferation and death is not independent. In healthy tissues, cell density homeostasis is achieved by compensating for cell death with an appropriate rate of cell proliferation. This process is known as “apoptosis-induced proliferation” (reviewed by Heitzer et al. (2) and Ryoo et al. (56)), but it is unclear how significant a role it plays in tumors. The consequences for ctDNA shedding could be straightforward, where increased cell death leads to increase cell birth and so on, and both processes result in increased ctDNA levels. However, positive feedback mechanisms like this may be tissue-dependent and could be dysregulated in cancer, complicating interpretation of ctDNA dynamics.

### Tumor Vasculature, Blood Vessel Proximity, and Hypoxia

Tumor vascularization and proximity to major blood vessels are also features of tumor physiology that might be expected to significantly impact ctDNA levels. Blood flow to a tumor is the direct means by which ctDNA enters circulation and it affects the metabolic activity of a tumor by providing oxygen and nutrients (57). As a tumor grows its vasculature becomes more irregular and dysfunctional leading to reduced oxygen levels, hypoxia and necrosis. Necrosis, reduced nutrient levels, and limited accesses to wider blood circulation all potentially effect ctDNA abundance in unique ways. Vasculature can also impact drug delivery and efficacy, which may also affect ctDNA shedding. It is unclear how much ctDNA abundance is dependent on direct access to blood vessels. Interstitial ctDNA is assumed to passively enter circulation through nearby blood vessels, but other processes like macrophage clearance of dead and dying cells (see “*Immune Response*” and “*Immunotherapy*” sections below) may also play a role in transporting ctDNA from areas with poor vasculature to the bloodstream (8, 25, 27).

Results from studies directly comparing tumor vascularization, angiogenesis, and ctDNA abundance, are inconsistent between studies. Post-excision pathology by Abbosh et al. (4) found lymphovascular invasion to be predictive of ctDNA detection in early-stage lung cancer. Two other studies in lung cancer found vascular invasion to be marginally (47), or not at all (46), correlated with ctDNA detection. Interestingly, when only looking at patients with EGFR mutations, ctDNA was significantly correlated with vascular invasion in the former study by Cho et al. (47). In liver cancer, microvascular invasion was correlated with preoperative ctDNA levels (58). In recent preliminary data collected from a large cohort of colorectal cancer (CRC) patients, ctDNA was found to be strongly associated with lymphovascular invasion (59). In neurological malignancies, which typically have less detectable ctDNA, Nabavizadeh et al. (60) found that tumor vessel size was correlated with detectable ctDNA. Notably, previous work by the same group and others found that the amount of microvascular proliferation was not significantly correlated to ctDNA in glioblastoma (GBM) specifically (61, 62). The nature of such studies makes it challenging to discern if these correlations are independent of tumor stage and volume. Proving a causal link may only be possible with further evaluation of preclinical models, tumor pathology, and imaging.

Hypoxia is in many ways a measure of tumor cell access to functional vasculature (63). As a tumor grows, cells become more isolated from functional vasculature, despite upregulated angiogenesis that is characteristic of many cancers. This process selects for cells that are more tolerant of low-oxygen conditions while the remaining population become necrotic (64). There is a clear link between hypoxia and necrosis and some studies have suggested that ctDNA is primarily derived from necrotic processes (1, 35, 65, 66). This suggests that as a tumor grows and vasculature becomes more distant and dysfunctional, wider ctDNA abundance could either increase due to further necrosis, or decrease due to reduced access to that vasculature. Since both forces are not equal in all tumors, the overall effect on ctDNA levels from this process may not be neutral. *In vitro* experiments with CRC cells have found that hypoxic conditions induced cell-free DNA production during the first 24 h but decreased dramatically over the following 48–72 h (37). These results are also consistent with previous findings in both tumor-injected and tumor-free mice where hypoxia induced cell-free DNA release (67). Deprivation of the metabolite, folate, has been found to induced double-strand DNA breaks and mis-segregation events, which may also lead to ctDNA shedding in nutrient-starved tumors as well (68).

### Organ Encapsulation

The free movement of cell-free DNA between tissue and blood may be restricted in some organs. Blood-tissue barriers have been identified throughout the body, such as the thymus, testes, retina, and intestines, but it is unclear what role they might play in cell-free DNA exchange (69). The blood-brain barrier (BBB) is often cited as the primary reason that neurological malignancies, particularly gliomas, produce less detectable ctDNA than other cancer types (29). In a 2018 review on ctDNA kinetics, Khier and Lohan speculate that physiological barriers, like the BBB, restrict the movement of cell-free DNA throughout the body while also acknowledging the exception of placental cell-free DNA, which has been shown to move quite freely throughout the mother (8, 70). Notably, disruption of the BBB that results in increased permeability and risk of metastasis also resulted in increased levels of ctDNA in patients with GBM (60). Several studies have shown that disrupting the BBB in animal models using focused ultrasound techniques leads to increase blood levels of ctDNA and other biomarkers (71, 72). Therefore, it is possible that changes in tissue-blood barrier permeability, particularly during treatment, might significantly affect ctDNA dynamics.

### Immune Response

Although there may be a significant role for inflammation and infection (e.g., sepsis) in cell-free DNA release, this review is primarily interested in the extent to which they directly impact ctDNA release from cancer cells. Early studies exploring the origins of cell-free DNA found that macrophages may play a significant role in cell-free DNA release though phagocytosis of dead and dying cells (73). Phagocytes have been shown to digest apoptotic cells and release the resulting cell debris and fragmented DNA (24, 73, 74). In the GBM study mentioned earlier, ctDNA levels were strongly associated with the density of macrophages around the tumor (60). In healthy individuals cell turnover is a tightly regulated process where apoptotic cells are quickly removed by phagocytes, however, this process appears to be dysfunctional in tumors resulting in excess cell debris (including DNA) that accumulates locally and in circulation (27, 66). The extent to which ctDNA levels might be directly affected by tumor cell targeting and/or clearance by immune cells is still an open question. Evidence for this phenomenon however, might be found in studies where ctDNA levels spike within 2 weeks of immune-therapy initiation in metastatic melanoma patients, if and only if, the tumors were responsive (75).

### Cell-Free DNA Clearance

Cell-free DNA digestion and clearance, whether achieved locally *via* phagocytosis or in circulation via the liver, spleen, and kidneys, is influenced by a number of factors (8, 27, 76, 77). As described above, cell-free DNA clearance *in situ* is potentially dependent on interstitial diffusion and the presence of phagocytic cells, however, once it is in circulation its half-life is determined by extracellular DNase activity and organ function (8). ctDNA half-life in the blood ranges from 30 to 120 min (27) making blood collection timing critical. The decreased levels of DNase activity observed in the blood of cancer patients potentially explains the accompanying increase in cell-free DNA levels from disruption of homeostasis (78, 79). Studies have also suggested that cell-free DNA clearance and half-life is dependent on proper liver and kidney function suggesting that treatment toxicity in cancer patients could affect ctDNA clearance rates and abundance (80, 81). The role of renal function in ctDNA clearance is not well understood, however, based on experiments assessing cell-free DNA levels in urine (27). The presence of cell-free DNA in urine implies involvement of the kidneys in clearance from circulation, however, patients with chronic renal failure were found not to have increased levels of cell-free DNA in their plasma (82). Methylation profiling has suggested that cell-free DNA present in urine is derived from white blood cells, kidney cells and urinary tract cells, but data from stem cell transplant patients found that the majority of this DNA was from the renal system itself and not plasma (83, 84).

## The Effect of Treatment on Circulating Tumor DNA Abundance

**FIGURE 2:**
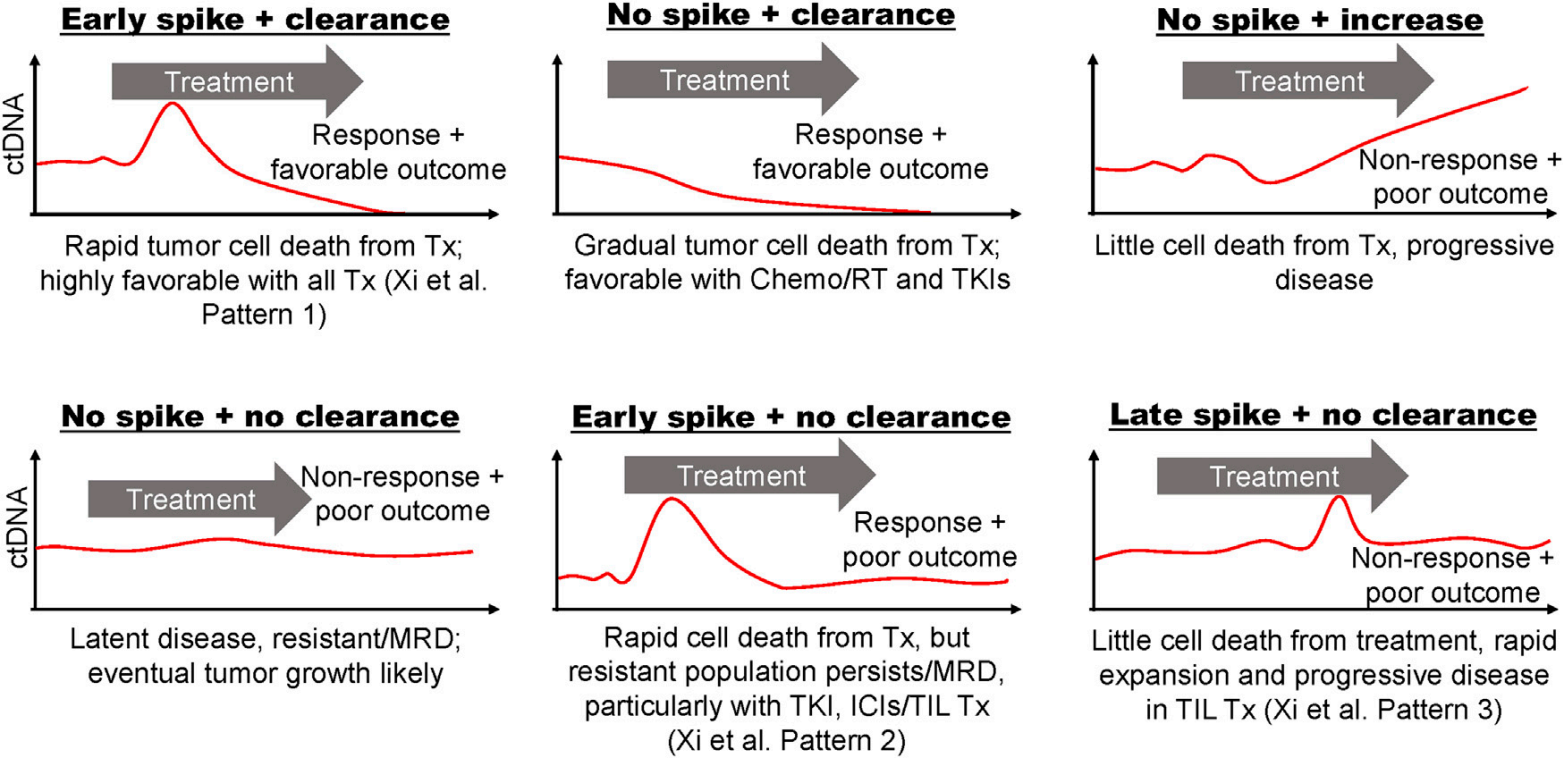
Hypothetical ctDNA dynamics from blood sampled frequently before, during, and after treatment. Characteristic ctDNA dynamics are depicted as observed in various studies throughout the text or otherwise hypothesized. The speculated driving tumor biology is described for each plot and example outcomes are stated. Response patterns defined by Xi et al. (14) for metastatic melanoma patients undergoing tumor infiltrating lymphocyte (TIL) immunotherapy are indicated when relevant. Tx, treatment; TKI, tyrosine kinase inhibitors; ICI, immune checkpoint inhibitors; MRD, molecular/minimal residual disease; ChemoRT, chemoradiation therapy.

The effect of treatment on tumor cell proliferation and death, and thus ctDNA dynamics, is dependent on its mechanism of action, efficacy, and tumor biology. Considering the factors described above that influence ctDNA abundance, it is not surprising that there are many discernable differences between the ctDNA dynamics of responders and non-responders during treatment. Predicting their timing and trajectories is not so simple, particularly when considering the short half-life of ctDNA. We might at least expect that ctDNA dynamics should reflect treatment response depending on the mechanism of action of a given treatment, but the timing of those effects is still unclear (Figure 2). The correlation between *in vitro* and *in vivo* models of treatment-induced cell death remains largely unclear, and is likely dependent on a variety of factors including the treatment and tissue of interest. Despite the paucity of data, it is possible that tumor cell death can occur within hours of treatment and therefore ctDNA levels may rapidly increase as well (36, 85–87). Serial ctDNA monitoring has been done with collection times ranging from minutes, hours or days after administration of treatment, to weeks and months. Some guidance may be gleaned from these studies, but the optimal time for sampling may be unique to cancer type and therapy and may need to be determined empirically. The following sections outline the expected effects of cancer treatment modalities on ctDNA dynamics, and what existing evidence, if any, tells about these hypotheses.

### Chemotherapy and Radiation

Cytotoxic chemotherapies and radiation therapy (RT) are often used independently or in combination as first-line treatment in many cancers. Chemotherapy agents function by disrupting mitosis or causing DNA damage leading to cell-cycle arrest, mitotic catastrophe, and apoptosis. Radiation therapy kills cells by DNA damage as well, but it also elicits an immune response and vascular damage, which can result in subsequent rounds of tumor cell death. Mitotic arrests and DNA damage are thought to cause tumor cell death within 6–72 h of administration *in vivo*, so detecting ctDNA shedding in response to effective treatment may require immediate sampling (87). Unfortunately, very few studies sample ctDNA within the first 72 h of chemotherapy. It has also been suggested that treatment-induced mitotic catastrophe can cause delays in cell death from chemotherapy and RT for up to a week (36, 62, 88). These various mechanisms of action may result in multiple shedding events, where one may be more informative about treatment response over another.

Limited studies assessing ctDNA levels immediately after treatment are conflicting. In castration-resistant prostate cancer patients receiving docetaxel-based therapy, early ctDNA levels were found to increase rapidly within 1 h of administration with a corresponding decrease in total cell-free DNA (89). This observation is consistent with increased tumor cell sensitivity to cytotoxic agents compared to healthy tissue. Contrary to these findings, however, CRC patients receiving FOLFOX did not exhibit a spike in ctDNA at any point within the first 48 h of treatment, despite high-resolution sampling at 3, 9, 18, 23, 26, 42, and 47 h (90). Another study involving metastatic CRC patients receiving FOLFIRI looked at ctDNA levels before and 7 days after each of the first two treatment cycles and again at progression (91). Interestingly, this study found that temporary increases in ctDNA while on treatment were predictive of progressive disease and worse survival rates, and suggested ctDNA monitoring within the first week of treatment to evaluate treatment efficacy. While ctDNA levels were decreased at the time of radiological assessment compared to baseline for all patient, patients with temporary spikes in ctDNA appeared to have more sustained ctDNA burden than those with favorable response (see Figure 2: “Early spike + no clearance”). Clonal composition was also found to vary during treatment suggesting a differential response to treatment among tumor cell subpopulations. Increases in cell-free DNA methylation levels of tumor suppressor genes (APC and RASSF1A) 24 h after receiving cisplatin-based chemotherapy were correlated with improved tumor response and overall outcome in advanced lung cancer patients (92). The same study showed that methylation levels of those genes in lung cancer cells also peaked 24 h after cisplatin exposure, however, it is unclear if the hypermethylated DNA was tumor-derived in patients. Notably, this study also found that elevated methylation of APC and/or RASSF1A in tumor-bearing mice were associated with tumor cell death as determined by biopsy shortly after treatment and blood collection. This finding might suggest that the methylated cell-free DNA originated from these dying tumor cells. In pancreatic cancer patients sampled for 4 weeks following treatment with gemcitabine, decreases in ctDNA were correlated with tumor response (93). In our work and others’, decreases in or complete clearance of ctDNA levels during low-resolution sampling (i.e., weeks to months) of neoadjuvantly-treated breast cancer patients were associated with pathological complete response at the time of surgery (9–11).

In patient cohorts receiving combination chemoradiation therapy (CRT), decrease and clearance of ctDNA after 3–4 weeks was associated with tumor response in oropharyngeal and lung cancer (94, 95). Studies employing high-frequency sampling at time points within hours of treatment are sparse, however, recent data from Breadner et al. (96) found that ctDNA abundance increased in 77% of stage III/IV non-small cell lung cancer patients shortly after receiving CRT with peak abundance observed 7 h after chemotherapy initiation and 2 days after the first fraction of radiation. Early spikes in ctDNA were seen in some patients receiving CRT for treatment of locally advanced head and neck cancer, but were not correlated with response (16). Rather, overall decreases in ctDNA at later time points, which were not unique to patients who had early peaks, were more predictive of outcome.

Some of the earliest observations of cell-free DNA by Leon et al. (97) occurred in patients receiving RT alone, finding that general cell-free DNA levels decreased after treatment. Aucamp et al. (24) speculate that the reason for this may have been the coincident destruction of phagocytes needed to generate cell-free DNA. Another consideration that may confound ctDNA measurement from irradiated tumors is that, although mitotic arrest and catastrophe are the primary means by which RT is thought to kill cancer cells, they have also been found to result in mis-segregation events (98, 99). As discussed above, this process can result in releasing of DNA from living cells, providing another potential source of ctDNA that does not coincide with cell death. A recent study found that irradiation of head and neck cancer and NSCLC cell lines induced cell-free DNA shedding after 6–24 h in culture (36). The same study found that ctDNA levels increased within 24 h and peaked 96–144 h after 20 Gy of irradiation in xenograft mouse models. Interestingly, the authors found that treatment-induced senescence that was overcome with the senolytic drug, Navitoclax, lead to apoptosis and increased ctDNA release. In human subjects, investigation of RT alone in NSCLC has shown mixed results. Walls et al. (100) found that 3 of 5 patients had decreased ctDNA levels 3-day after their first RT fraction, while the remaining 2 had increased levels of some tumor-derived variants, but not others. Preliminary data from our lab (101) and another study from Chen et al. (102) found that ctDNA levels were elevated 24–48 h after the first dose of stereotactic ablative radiotherapy. These three studies in NSCLC patients used varying doses of radiation per fraction (2.75, 12, and ∼12.5 Gy, respectively), which along with sampling time differences, may account for the discrepancy. Chaudhuri et al. (103) also reported that mid-RT ctDNA levels in 13 NSCLC patients were correlated with outcomes at 2 years. A recently published study by our lab found that a metastatic breast cancer patient had increased ctDNA levels while receiving palliative radiation therapy. Deep sequencing using a 53-mutation panel representative of both clonal and subclonal mutations, which were previously identified from WES of multiple tumors, revealed differential response in ctDNA levels for various subclones with sample collection every 48-hour during RT (104). Differential response in subclonal ctDNA abundance is suggestive of varying sensitivity in irradiated tumor cell subpopulations and/or an abscopal response. First observed by Dr. R.H. Mole in 1953, the abscopal effect is the shrinkage of a distant, untreated tumor in response to RT of another tumor. It is thought that the destruction of cells in the irradiated tumor elicits an immune response that affects the non-radiated tumors elsewhere in the body (105). In our case, radiation of a single lesion may have induced immune-mediated responses and ctDNA shedding from distant metastatic sites that harbored subclonal tumor cell populations. It is also worth noting that CTCs may also be sources of ctDNA and CTC release timing may also be similar to tumor ctDNA shedding (106). In preliminary data in head and neck cancer patients treated with RT, 3 of 11 patients had increased circulating tumor cell (CTC) counts after the first fraction of RT, and 5 of 6 patients had increases CTCs after 2 weeks into therapy.(107).

### Immunotherapy

ctDNA monitoring during treatment with immune checkpoint inhibitors (ICIs) has shown promise in multiple cancer types. A pan-cancer analysis done by Zhang et al. (17) found that changes in ctDNA levels during ICI treatment may be predictive of benefit. Patients in this study with increases in ctDNA levels during treatment had worse outcomes as compared to those that did not. Furthermore, patients with ctDNA clearance after detectable pre-treatment levels had the best progression-free and overall survival. A recent study by Herbretreau et al. (75) in patients with metastatic melanoma found that significant increases in ctDNA levels during the first 2–4 weeks of anti-PD1 (with or without anti-CTLA4) allowed early and highly-specific identification of treatment-resistant patients. Furthermore, ctDNA levels that rapidly decreased after starting PD-1 inhibitors were highly predictive of responses consistent with pseudoprogression (108, 109). When compared to changes in ctDNA levels later in treatment, regardless of early changes, increases beyond 12 weeks were not necessarily predictive of non-response, further suggesting that early sampling is more informative (110). In NSCLC patients treated with ICI, decreases in ctDNA 2 weeks after treatment initiation were strongly correlated with radiographic response and progression-free survival (111). A study investigating early response to tumor infiltrating lymphocyte (TIL) immunotherapy in metastatic melanoma patients identified three patterns of ctDNA dynamics that could be used to stratify patients by overall survival (14). Patients with an early spike in ctDNA within 5–10 days of treatment followed by clearance showed a statistically significant survival outcome over patients who had early peaks but latent ctDNA burden, or no peaks with or without clearing (see Figure 2). The study’s authors speculate that early spike it ctDNA was in part due to the newly-transferred lymphocytes “identifying their targets and are effective in killing [them].”

### Targeted Therapy

Given the clinical implications of tumor heterogeneity, one of the most significant unanswered questions in ctDNA analysis is whether ctDNA observed during therapy is more representative of resistant or responsive tumor cell populations. In a study of lung cancer patients undergoing EGFR tyrosine kinase inhibitor (TKI) therapy, ctDNA sampled 2 weeks after treatment initiation revealed activating mutations not previously detected in the tumor biopsies (112). Another study of lung cancer patients on TKIs found that clearing of ctDNA within days of treatment was associated with response, whereas sudden increases in ctDNA load later in treatment correlated with rapid tumor progression and poor outcome (113). In lung cancer patients receiving either anti-EGFR or HER2 therapies, increases in ctDNA abundance were observed within 4–12 h after initiation of treatment while total cell-free DNA was relatively constant (86). Phallen and coauthors point out that this timeframe is consistent with other studies in which apoptosis is observed *in vitro* within 6–48 h of treatment with EGFR TKIs. Patients in this study with an initial radiographic response all had ctDNA abundance eventually decrease by more than 95% within the first 19 days of treatment. Interestingly, baseline levels in a study of ALK-fusion positive lung cancer patients, pre-treatment ctDNA levels were not correlated with treatment response, yet changes in ctDNA during treatment with ALK TKIs were associated with progression (114).

Increases in ctDNA abundance of therapy-sensitive clones corresponds with response, however, increases in ctDNA abundance of therapy-resistant clones can also portend clinical progression (2). Outgrowth of subclonal tumor cell populations that are resistant to targeted therapy can be directly observed in allele-specific ctDNA dynamics. For example, genomic changes conferring resistance to targeted therapy in prostate cancer patients were detected by increasing fractions of the resistance-associated allele in several studies (115, 116).

## Discussion

Host physiology and tumor biology affect ctDNA abundance while changes in ctDNA levels during treatment may indicate disease response. Cancer type and stage appear to have the most dramatic impact on ctDNA abundance, and significant decreases in, or clearance of ctDNA early in treatment seems to be predictive of response and improved outcomes. The association of treatment response and overall decreases in ctDNA levels during treatment is consistent with the hypothesis that tumor burden and tumor growth rate are reflected in ctDNA dynamics. Very early changes (1–3 h) in ctDNA levels have been hypothesized to reflect treatment response as well, but this appears to be less generalizable. For example, we might expect effective chemotherapy to induce ctDNA shedding immediately, and early, high-frequency sampling to detect it, yet observations between NSCLC, CRC and prostate cancer patients sampled within 1–3 h of chemotherapy were inconsistent (89, 90, 117). Unfortunately, there are a lack of studies sampling within this timeframe. Immunotherapy may be less fast-acting than chemotherapy given the time required for the body to prepare a successful immune response. PET/CT imaging has shown tumor responses with 4–6 weeks of treatment with ICIs in melanoma patients (118). Sample collection at 2 weeks following treatment found changes in ctDNA that correlated with outcome in ICI therapy of melanoma patients, but earlier time points were not collected (75). It possible that changes in ctDNA in response to treatment existed sooner, again, earlier, high-frequency sampling is needed to test such hypotheses. TKI-induced cell death appears to occur within 6–48 h of exposure *in vitro* (85); a similar timeframe as cell death from cytotoxic agents. Evidence presented in this review suggests that ctDNA dynamics might reflect TKI-induced cell death in this timeframe in NSCLC patients more consistently than during chemotherapy.

One potential use of ctDNA monitoring during treatment that has been explored by our lab and others (96, 101), is to induce ctDNA shedding from either inaccessible tumors or suspicious lesions for evaluation. Radiation treatment seems particularly suited for this task, however any method of perturbation that elicits ctDNA shedding could be used. For example, such approaches could improve the detection rates of ctDNA assays like CAPP-Seq and Lung-CLiP (45) in lung cancer patients or where low-dose CT is already in use for screening high-risk populations. Compression of breast tissue during mammography has been shown to temporarily increase ctDNA abundance, which could be leveraged for non-invasive biopsy or early detection (44). Other work has explored the use of ultrasound to elicit better movement of blood biomarkers across the BBB in preclinical brain tumor models (71, 72). Again, a clear understanding of early ctDNA dynamics in response to tumor perturbations is crucial before such approaches can be implemented in the clinic.

Variability in ctDNA measurements between patients and studies has been a challenge for serial monitoring. Efforts have been made to assess the biological variability of both cell-free DNA and ctDNA between measurements taken over short intervals (31, 119). Several groups have attempted to standardize criteria for evaluating differences between pre-treatment and on-treatment ctDNA levels. O’Leary et al. (120) created a “circulating tumor DNA ratio” or CDR, defined simply as the ratio of on-treatment to pre-treatment levels, to evaluate treatment response in metastatic breast cancer. Herbreteau, Kruger et al. (2018 and 2021) (75, 93) defined a “quantitative biological response and progression criteria” where patients were stratified by increases or decreases in ctDNA during treatment as compared to baseline. This approach also recognized the variability in accuracy at each time point when evaluating significance between measurements at different time points. Out of similar concern, our lab has also developed a Bayesian approach for testing statistical significance between ctDNA mutations in NGS data from serial collections (10). Given the variability in coverage of NGS data, which determines the limit of detection, the allele-specific background error rate, and the stochastic nature of mutant read detection by NGS, sophisticated methods may be required to account for these uncertainties in ctDNA evaluation.

Finally, attempts to model ctDNA shedding have shown promise in predicting ctDNA abundance based on tumor size and growth rate. Avanzini et al. (48) presented a stochastic mathematical framework based on observations in lung cancer patients which could extrapolate ctDNA copy counts using tumor cell proliferation rate, death rate, shedding probability, clearance rate, and starting tumor volume. Such modeling can reveal unexpected behavior that may be informative of real-world scenarios. For example, in simulations the authors unexpectedly found that a slow growing cancer generated more ctDNA molecules than a faster-growing cancer of the same size when the faster growth was achieved by proportional increases and decreases in birth and death rates, respectively. However, if a faster growth rate is achieved by increased birth rate and a stable death rate, the difference in ctDNA release was negligible. By integrating these variables over time, similar models might be useful in predicting changes in ctDNA abundance from changes in birth and death rates resulting from treatment.

## Conclusion

The utilization of ctDNA in assessing treatment response will require a better understanding of the biological factors involved. We believe that ctDNA monitoring has the potential to truly revolutionize personal medicine in cancer care but there remain significant challenges that must first be overcome.
