# Corrigendum: Blood, toil, and taxoteres: Biological determinants of treatment-induced ctDNA dynamics for interpreting tumor response

**DOI:** 10.3389/pore.2023.1611133

**Published:** 2023-03-09

**Authors:** Christopher T. Boniface, Paul T. Spellman

**Affiliations:** ^1^ Knight Cancer Institute, Oregon Health & Science University, Portland, OR, United States; ^2^ Cancer Early Detection Advanced Research Center, Knight Cancer Institute, Oregon Health & Science University, Portland, OR, United States

**Keywords:** biomarkers, liquid biopsy, circulating tumor DNA, ctDNA, tumor growth, treatment response

In the published article, there were two typographical errors in the article title. Instead of “Blood, toil, and taxoteres: Biological determinates of treatment-induce ctDNA dynamics for interpreting tumor response.” It should be “Blood, toil, and taxoteres: Biological determinants of treatment-induced ctDNA dynamics for interpreting tumor response.”

The authors apologize for this error and state that this does not change the scientific conclusions of the article in any way. The original article has been updated.

